# PGAM2 Regulates Sepsis-Induced Diaphragmatic Atrophy via the JAK2/STAT3 Pathway

**DOI:** 10.3390/biomedicines14051075

**Published:** 2026-05-09

**Authors:** Yun Chu, Xinrun Yuan, Xiaopo Gao, Jinlong Luo

**Affiliations:** 1 Department of Intensive Care Medicine, Tongji Hospital, Tongji Medical College, Huazhong University of Science and Technology, Wuhan 430030, China; b2025001062@student.pumc.edu.cn (Y.C.); m202476657@hust.edu.cn (X.Y.); m202376666@hust.edu.cn (X.G.); 2 Department of Emergency, Tongji Hospital, Tongji Medical College, Huazhong University of Science and Technology, Wuhan 430030, China

**Keywords:** sepsis, diaphragmatic dysfunction, muscle atrophy, JAK2/STAT3

## Abstract

**Background/Objectives**: Sepsis-induced systemic inflammation often leads to diaphragmatic dysfunction and muscle atrophy, contributing to impaired respiratory function. Phosphoglycerate mutase 2 (PGAM2), a key enzyme in glycolysis, plays a significant role in muscle energy metabolism but has not been previously linked to sepsis-induced diaphragmatic dysfunction. This study aims to investigate the role of PGAM2 in sepsis-induced diaphragmatic atrophy and its underlying mechanisms. **Methods**: A murine sepsis model was established using cecal ligation and puncture (CLP) in C57BL/6 mice. Body and diaphragm weights, along with muscle fiber cross-sectional areas, were measured. PGAM2 expression was evaluated using immunofluorescence, Western blotting, and real-time quantitative polymerase chain reaction (RT-qPCR). In vitro, C_2_C_12_ myotubes were treated with tumor necrosis factor alpha (TNF-α), and PGAM2 expression was manipulated via small interfering RNA (siRNA) knockdown and plasmid overexpression. Atrophy markers (MuRF1, MAFbx/atrogin-1) and JAK2/STAT3 pathway activation were assessed. **Results**: CLP induced significant diaphragmatic atrophy, as reflected by an approximately 38% reduction in diaphragm weight and an approximately 37% decrease in muscle fiber cross-sectional area compared with the sham group. In contrast, PGAM2 protein expression was increased by approximately 105% in septic diaphragms. PGAM2 expression was also significantly elevated in TNF-α-treated myotubes. PGAM2 knockdown resulted in reduced MuRF1 and MAFbx expression, attenuating myotube atrophy, while PGAM2 overexpression exacerbated atrophy. Moreover, PGAM2 knockdown suppressed activation of the JAK2/STAT3 signaling pathway. **Conclusions**: These findings demonstrate that PGAM2 contributes to sepsis-induced diaphragmatic atrophy through the activation of the JAK2/STAT3 signaling pathway. PGAM2 may therefore serve as a potential therapeutic target for sepsis-associated diaphragmatic dysfunction.

## 1. Introduction

Sepsis induces a systemic inflammatory response marked by elevated pro-inflammatory cytokines such as IL-1β, IL-6, and TNF-α, which impair skeletal muscle contractility [1,2,3,4,5]. In parallel, circulatory disturbances, including hypotension and impaired perfusion, reduce oxygen delivery to the diaphragm, leading to tissue hypoxia [5,6,7]. These combined effects contribute to respiratory failure, often necessitating mechanical ventilation [7]. However, while lifesaving, mechanical ventilation can exacerbate diaphragmatic injury by altering protein metabolism, enhancing oxidative stress, and impairing mitochondrial function [8,9]. Among the molecular pathways involved, the ubiquitin–proteasome system (UPS) plays a central role in mediating protein degradation and muscle atrophy [10]. In sepsis, the UPS is activated by cytokines, oxidative stress, and metabolic disturbances, accelerating muscle protein degradation [11]. The E3 ligases MuRF1 and MAFbx/, both upregulated in sepsis, promote breakdown of myosin and other muscle proteins [12].

Phosphoglycerate mutase (PGAM) is a glycolytic enzyme that catalyzes the interconversion of 3-phosphoglycerate and 2-phosphoglycerat [13,14]. Zebrafish lacking PGAM2 exhibit muscle atrophy with reduced fiber size, suggesting its key role in muscle growth [15]. Importantly, PGAM2 function is not determined solely by its native form but is further modulated through post-translational modifications [16,17]. For instance, SUMO-modified PGAM2 inhibits myogenic differentiation in C_2_C_12_ cells [18].Beyond its glycolytic role, PGAM2 has been implicated in pathological processes: its constitutive overexpression reduces cardiac stress tolerance in heart failure [19], and its expression is strongly regulated by TNF-α during skeletal muscle differentiation, linking it to inflammatory muscle atrophy [20,21]. These findings suggest that PGAM2 alterations under inflammatory conditions may contribute to muscle dysfunction, although its precise role in inflammation and myopathy remains unclear.

Recent evidence suggests that PGAM2 also exerts non-glycolytic regulatory functions. By binding to the 14-3-3 zeta protein, PGAM2 facilitates interaction with phosphorylated BAD, thereby activating BCL-xL and inhibiting apoptosis [22]. In pathological cardiac hypertrophy, PGAM2 competes with the E3 ubiquitin ligase SYVN1 for HSP90 binding, thereby suppressing the mTOR/IKKα pathway and limiting hypertrophy [23]. Moreover, the related isoform PGAM1 directly interacts with the JAK2/STAT3 pathway in other tissues [24]. Given that PGAM2 is more abundant in skeletal muscle, it is plausible that it may similarly regulate JAK2/STAT3 signaling, a central mediator of sepsis-induced muscle atrophy. However, whether PGAM2 participates in this pathway, and thus contributes to inflammation-associated diaphragmatic atrophy, remains unknown.

To address this knowledge gap, we used a CLP mouse model of sepsis together with a TNF-α-stimulated C_2_C_12_ myotube model to examine the role of PGAM2 in diaphragmatic atrophy. Our study demonstrates that PGAM2 contributes to sepsis-induced diaphragm atrophy and reveals the underlying molecular mechanisms, offering new insight into the pathogenesis of sepsis-related respiratory muscle failure and highlighting PGAM2 as a potential therapeutic target.

## 2. Materials and Methods

Fetal bovine serum was obtained from Yikesai (Suzhou, China), and horse serum from Soleibao (Beijing, China). BCA protein assay and CCK-8 kits were purchased from Zigong (Shanghai, China). Dulbecco’s modified Eagle medium (DMEM), phosphate-buffered saline (PBS), and protease/phosphatase inhibitors were obtained from MedChem Express (Monmouth Junction, NJ, USA). Tumor necrosis factor-α (TNF-α) was purchased from Sigma-Aldrich (St. Louis, MO**,** USA). TRIzol reagent for RNA extraction was obtained from Takara Clontech (Beijing, China). Antibodies against phosphorylated JAK2 (p-JAK2), total JAK2, phosphorylated STAT3 (p-STAT3), and total STAT3 were obtained from Cell Signaling Technology (Danvers, MA, USA). Antibodies against Atrogin-1, MuRF1, Laminin α2, and MyHC were purchased from Santa Cruz Biotechnology (Dallas, TX**,** USA). Unless otherwise specified, additional reagents were obtained from Servicebio (Wuhan, China).

### 2.1. Experimental Design and Treatment

SPF-grade male C57BL/6 mice (Bainet Biotechnology Co., Ltd., Suzhou, China) were housed under standard conditions (22 ± 2 °C, 12 h light/dark cycle, 50 ± 10% humidity) with free access to food and water. Animals were randomly assigned to the sham or CLP group. Body weight was monitored before surgery, daily after surgery, and at sacrifice. After anesthesia with intraperitoneal pentobarbital sodium (30–50 mg/kg; Sigma, St. Louis, MO, USA), CLP was performed as illustrated in the schematic diagram. Briefly, the cecum was exposed through a midline abdominal incision, ligated at the distal portion, punctured with a sterile needle, and gently squeezed to extrude a small amount of fecal content before being returned to the abdominal cavity. The abdomen was then closed in layers. In the sham-operated group, mice underwent the same anesthesia and laparotomy procedures, and the cecum was exteriorized and gently manipulated but was not ligated or punctured before being returned to the abdominal cavity. Sham-operated mice received the same wound closure and postoperative care as CLP mice. After surgery, mice received postoperative analgesia and were monitored for 72 h. On postoperative day 3, mice were euthanized with an overdose of 1% pentobarbital sodium, and diaphragms were collected for analysis.

**Figure 1:**
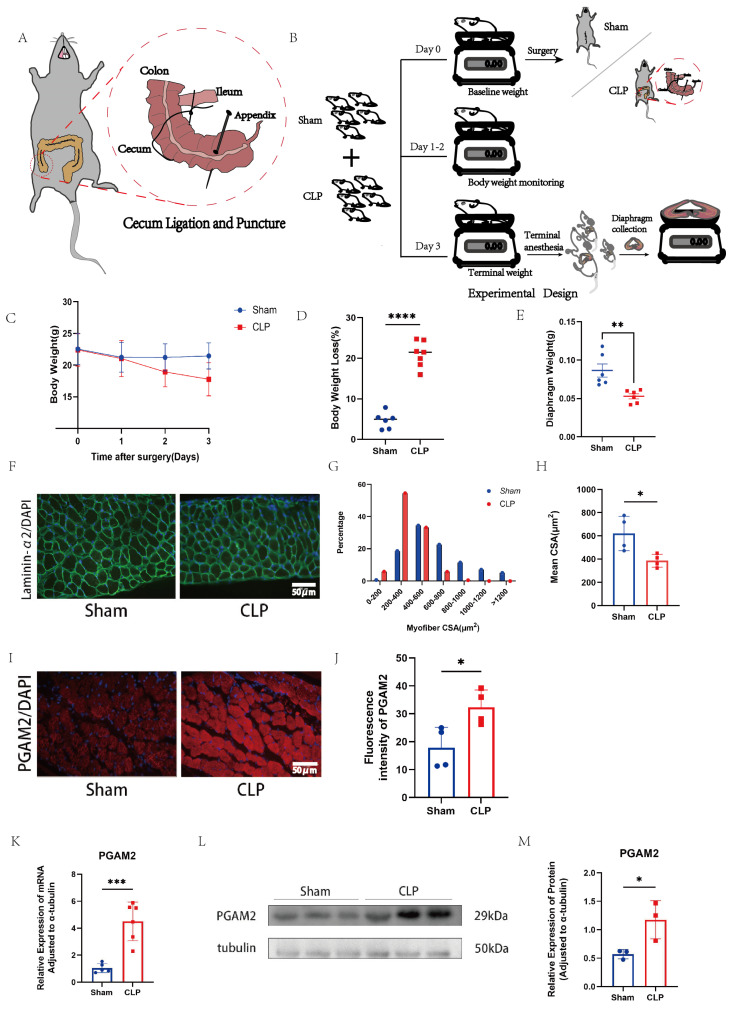
Sepsis-induced diaphragmatic atrophy is associated with upregulation of PGAM2. (**A**) Schematic representation of the cecal ligation and puncture (CLP) procedure. (**B**) Experimental design of the CLP mouse model. (**C**) Time-course of body weight following CLP or sham operation *(n* = 6–7). (**D**) Percentage loss of body weight 3 days after surgery (*n* = 6–7). (**E**) Diaphragm mass on post-operative day 3 (*n* = 5–6). (**F**) Representative immunofluorescence micrographs of diaphragm cross-sections. Laminin-α2 (green) delineates the basal lamina of muscle fibers; nuclei are counter-stained with DAPI (blue). (**G**) Frequency distribution of fiber CSA in sham and CLP groups (*n* = 4). (**H**) The mean diaphragm fiber cross-sectional area (*n* = 4). (**I**) Immunofluorescence images showing PGAM2 (red) and DAPI (blue) in diaphragm tissue. (**J**) Quantification of PGAM2 fluorescence intensity (*n* = 4). (**K**) Relative PGAM2 mRNA abundance in diaphragm homogenates (*n* = 5). (**L**,**M**) Representative immunoblots and densitometric quantification of PGAM2 protein in sham and CLP mice (*n* = 3). Scale bar = 50 µm. All data are presented as mean ± SEM. * *p* < 0.05, ** *p* < 0.01, *** *p* < 0.001, **** *p* < 0.0001 versus the corresponding control.

For the analyses shown in Figure 1, the number of animals included in each experiment varied slightly because some samples were excluded from specific analyses due to technical issues during tissue processing, including inadequate fixation, sectioning artifacts, or poor staining quality. No samples were excluded based on the experimental outcomes. Therefore, the final sample size was not fully balanced between groups for some experiments. Specifically, 6–7 mice per group were used for body weight loss and body weight time-course analyses; 5–6 mice per group were used for diaphragm mass measurement; 4 mice per group were used for fiber cross-sectional area frequency distribution and mean diaphragm fiber cross-sectional area analyses; 4 mice per group were used for PGAM2 immunofluorescence intensity analysis; 5 mice per group were used for PGAM2 mRNA analysis; and 3 mice per group were used for PGAM2 protein analysis by immunoblotting. The exact sample size for each experiment is indicated in the corresponding figure legends.

### 2.2. Surgical Process and Tissue Sampling

Sepsis was induced by cecal ligation and puncture (CLP) as previously described [25]. Briefly, under anesthesia, a midline laparotomy was performed to expose the cecum, which was ligated at half its length and punctured with a 21-gauge needle. A small amount of fecal content was extruded before the cecum was returned to the peritoneal cavity, and the incision was closed in two layers (Figure 1A). For diaphragm collection, mice were deeply anesthetized and placed supine on the operating table. A midline incision was extended from the abdomen to the costal margin and bilaterally along the rib arch. The thoracic cavity was opened to expose the heart, the right atrial appendage was incised, and physiological saline was perfused through the left ventricle to clear blood from the diaphragm. The intact diaphragm was then excised, rinsed, and weighed.

### 2.3. Immunofluorescence Staining

Approximately one-third of the fresh diaphragm was embedded in OCT compound and snap-frozen in liquid nitrogen. Cryosections of the diaphragm were cut at a thickness of 8–10 μm using a cryostat (Leica, Wetzlar, Germany). To ensure consistency in histological analysis, sections were obtained from the middle portion of the costal region of the diaphragm, which was used as the anatomical reference region for all animals.

For immunofluorescence staining, frozen diaphragm sections and C_2_C_12_ myotubes grown on coverslips were fixed with 4% paraformaldehyde for 15 min at room temperature and washed three times with PBS. Samples were blocked with 5% bovine serum albumin (BSA) for 1 h at room temperature and incubated with primary antibodies at 4 °C overnight. The primary antibodies included rabbit anti-PGAM2 antibody (Abcam, Cambridge, UK, ab187147, 1:1000), mouse anti-laminin-α antibody (Santa Cruz Biotechnology, Dallas, TX, USA, sc-59854, 1:1000), and mouse anti-MYH antibody (B-5) (Santa Cruz Biotechnology, sc-53089, 1:1000), a pan-myosin heavy chain antibody that does not distinguish between different muscle fiber types. After washing with PBS, samples were incubated with fluorescence-conjugated secondary antibodies, including goat anti-mouse IgG (Servicebio, Wuhan, China, GB21301, 1:100) and goat anti-rabbit IgG (Servicebio, Wuhan, China, GB21303, 1:100), in the dark for 1 h at room temperature. Nuclei were counterstained with DAPI for 5 min. Images were acquired using an Olympus BX51 fluorescence microscope (Tokyo, Japan) under identical exposure settings within each experiment.

For diaphragm sections, fluorescence images were acquired from the middle costal region of the diaphragm for each mouse. All images were captured at the same magnification. Laminin immunofluorescence staining was used to delineate the sarcolemma and muscle fiber boundaries for cross-sectional area (CSA) analysis. For each mouse, at least four immunofluorescence-stained sections were analyzed, and three fields of view were captured from the middle costal region of each section. Approximately 200 muscle fibers were measured per section. Obliquely sectioned, damaged, or poorly defined fibers were excluded from the analysis. Muscle fiber CSA was quantified using Fiji (a distribution of ImageJ, National Institutes of Health, USA), and the mean value from each mouse was used as one independent biological replicate for statistical analysis. For PGAM2 fluorescence intensity analysis, whole-image analysis was performed using ImageJ. The same thresholding and background subtraction procedures were applied to all images within the same experiment.

For C_2_C_12_ myotube analysis, three independent biological experiments were performed. In each independent experiment, multiple randomly selected non-overlapping microscopic fields were imaged, and myotube diameters were measured using ImageJ. The average myotube diameter from each independent experiment was used as one biological replicate for statistical analysis. Individual myotube measurements are shown in the scatter plots to illustrate data distribution, whereas statistical comparisons were performed using the independent biological replicates.

### 2.4. Cultivation of Escherichia Coli and Plasmid Cultivation

LB agar plates were prepared by dissolving 10 g LB powder and 1 g agarose in 100 mL distilled water, sterilized, cooled to approximately 50 °C, and supplemented with ampicillin (100 µg/mL). Escherichia coli harboring the PGAM2 plasmid were streaked and incubated at 37 °C for 18 h. Single colonies were inoculated into LB broth and cultured at 37 °C with shaking (200 rpm) for 16–18 h. Bacterial pellets were collected by centrifugation (5000 rpm, 5 min), and plasmids were isolated using a commercial kit (TianGen, Beijing, China). DNA concentration and purity were measured with a microspectrophotometer.

### 2.5. Cell Culture and Cell Viability Assay

The C_2_C_12_ mouse myoblast cell line (Bioxay, Beijing, China) was maintained in high-glucose DMEM supplemented with 10% fetal bovine serum at 37 °C in a humidified 5% CO_2_ incubator. Cells at ~80% confluence were transfected with siRNA (2 μL) or plasmid DNA (2 μg) using transfection reagent diluted in 100 μL PBS. For PGAM2 knockdown, the siRNA target sequence was 5′-CTGGTTTGATGCAGAGCTGAGTGAGAAGGGAGCAGAGGAGGCCAAGCGGGGGGCCACCGCTATCAAAGATGCCAAGATAGAG-3′. After 6–12 h, cells were switched to differentiation medium (DMEM with 2% horse serum). TNF-α (PeproTech, Cranbury, NJ, USA) was administered 12 h post-transfection, and proteins were harvested 24 h later for analysis.

For viability assays, C_2_C_12_ myotubes were seeded in 96-well plates (100 μL/well) and differentiated for 3 days. Cells were then treated with TNF-α at graded concentrations (0.001–10 μM) for 24–72 h. Cell viability was assessed using the CCK-8 kit according to the manufacturer’s instructions, and absorbance was measured at 450 nm. Each condition was assayed in triplicate.

### 2.6. Protein Immunoblotting

Protein lysates were prepared in RIPA buffer (Servicebio, Wuhan, China) supplemented with protease and phosphatase inhibitors (100:1:1). Cells were scraped or tissues were homogenized, and lysates were centrifuged at 12,000 rpm for 15 min at 4 °C to obtain supernatant proteins. Samples were mixed with 5 × SDS-PAGE loading buffer at a ratio of 4:1 and denatured at 95 °C for 5 min before separation by SDS-PAGE. The 5 × SDS-PAGE loading buffer contained 250 mM Tris-HCl (pH 6.8), 10% SDS, 50% glycerol, 0.5% bromophenol blue, and 5% β-mercaptoethanol. Proteins were transferred to PVDF membranes (Millipore, Burlington, MA, USA), blocked with 5% BSA, and incubated with primary antibodies overnight at 4 °C, followed by secondary antibody incubation for 1 h at room temperature. Immunoreactive bands were visualized using an enhanced chemiluminescent (ECL) substrate and captured with the GeneSys imaging system (Cambridge, UK).

### 2.7. Real-Time Quantitative Polymerase Chain Reaction (RT-qPCR)

Total RNA was extracted from cells or tissues using Trizol reagent (Takara, China) according to the manufacturer’s instructions. RNA concentration and purity were determined with a microspectrophotometer, and complementary DNA (cDNA) was synthesized using a reverse transcription kit (Takara, China). RT-qPCR wsas performed with SYBR Green Master Mix (Takara, China) on a real-time PCR system (Applied Biosystems, Waltham, MA, USA). β-actin was used as the internal control. Relative mRNA expression levels were calculated using the 2^−ΔΔCt^ method, with primer sequences listed in Table 1.

### 2.8. Data Analysis

All data were analyzed using GraphPad Prism 9.0. Results are expressed as mean ± SEM. For normally distributed data with equal variance, a *t*-test was applied between two groups; for non-normal data, the Kruskal–Wallis test was used. Statistical significance was set at *p* < 0.05.

## 3. Results

### 3.1. Sepsis Induces Diaphragm Atrophy of Mouse and Upregulates PGAM2 Expression

Cecal ligation and puncture (CLP) surgery was performed to establish a murine model of sepsis (Figure 1A), and the overall experimental design is illustrated in Figure 1B. Compared with Sham controls, CLP mice exhibited marked postoperative body weight loss over time (Figure 1C), with a significantly higher percentage of body weight loss at postoperative day 3 (Figure 1D). Accordingly, diaphragm mass was significantly reduced in CLP mice (Figure 1E). Immunofluorescence analysis of diaphragm cross-sections using laminin-α2 staining revealed obvious myofiber atrophy in septic mice (Figure 1F), as indicated by a left-shifted distribution of myofiber cross-sectional area (CSA, Figure 1G) and a significantly smaller mean CSA (Figure 1H; * *p* < 0.05). These results indicate that sepsis induces substantial diaphragmatic atrophy. To further explore molecular alterations associated with this phenotype, we examined PGAM2 expression in the diaphragm. Immunofluorescence staining demonstrated enhanced PGAM2 expression in CLP diaphragms (Figure 1I), which was confirmed by quantification of fluorescence intensity (Figure 1J). To further validate this finding, PGAM2 mRNA and protein levels were measured in diaphragms from CLP and Sham mice. Consistently, RT-qPCR confirmed significant upregulation of PGAM2 mRNA in CLP mice (Figure 1K), and Western blot assays verified increased PGAM2 protein expression (Figure 1L,M).

Collectively, these findings indicate that CLP-induced sepsis causes diaphragmatic atrophy and is associated with elevated PGAM2 expression.

### 3.2. TNF-α-Induced C_2_C_12_ Myotubular Atrophy and Increased Expression of PGAM2

**Figure 2:**
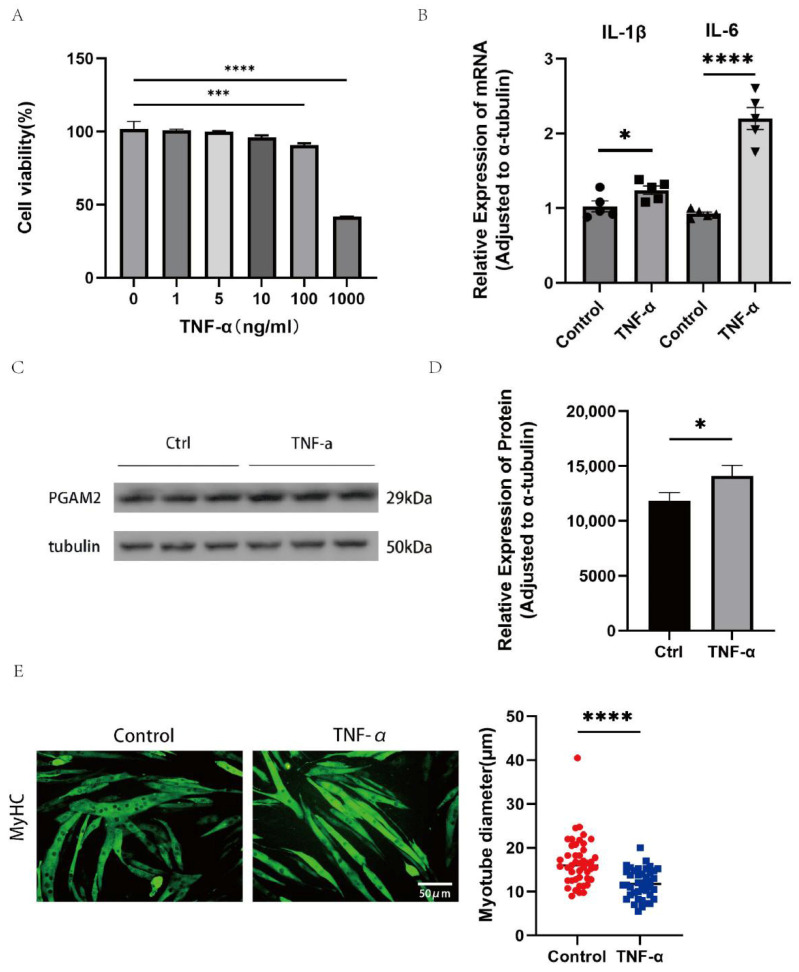
TNF-α increases PGAM2 expression and promotes myotube atrophy in C_2_C_12_ myotubes. (**A**) Cell viability of C_2_C_12_ myotubes treated with increasing concentrations of TNF-α for 24 h. (**B**) Relative mRNA levels in myotubes treated with or without TNF-α (100 ng mL^−1^). (**C**,**D**) PGAM2 protein expression in control and TNF-α–treated C_2_C_12_ cells *(n* = 3). (**E**) immunofluorescence images of myotubes in control and TNF-α–treated groups. Scale bar = 50 µm. * *p* < 0.05, *** *p* < 0.001, **** *p* < 0.0001.

To determine the appropriate concentration of TNF-α for cell stimulation, C_2_C_12_ cells were treated with different concentrations for 24 h, and cell viability was measured using a CCK-8 assay (Figure 2A). Cell viability decreased significantly at 100 ng/mL and 1000 ng/mL compared with the control group, while lower concentrations had little effect. Therefore, 100 ng/mL TNF-α was chosen for subsequent experiments to avoid excessive cytotoxicity in the whole experiments. Quantitative PCR showed that TNF-α treatment significantly increased the mRNA expression of IL-1β and IL-6 compared with the control group (Figure 2B), indicating successful induction of inflammation. Western blot analysis confirmed that PGAM2 protein levels were higher in TNF-α–treated cells (Figure 2C,D). Immunofluorescence staining of MyHC revealed that TNF-α exposure reduced myotube diameter (Figure 2E). Together, these results show that TNF-α stimulation creates an inflammatory muscle environment and increases PGAM2 expression, which may contribute to muscle atrophy.

### 3.3. PGAM2 Knockdown Relieves TNF-α-Induced Myotube Atrophy

**Figure 3:**
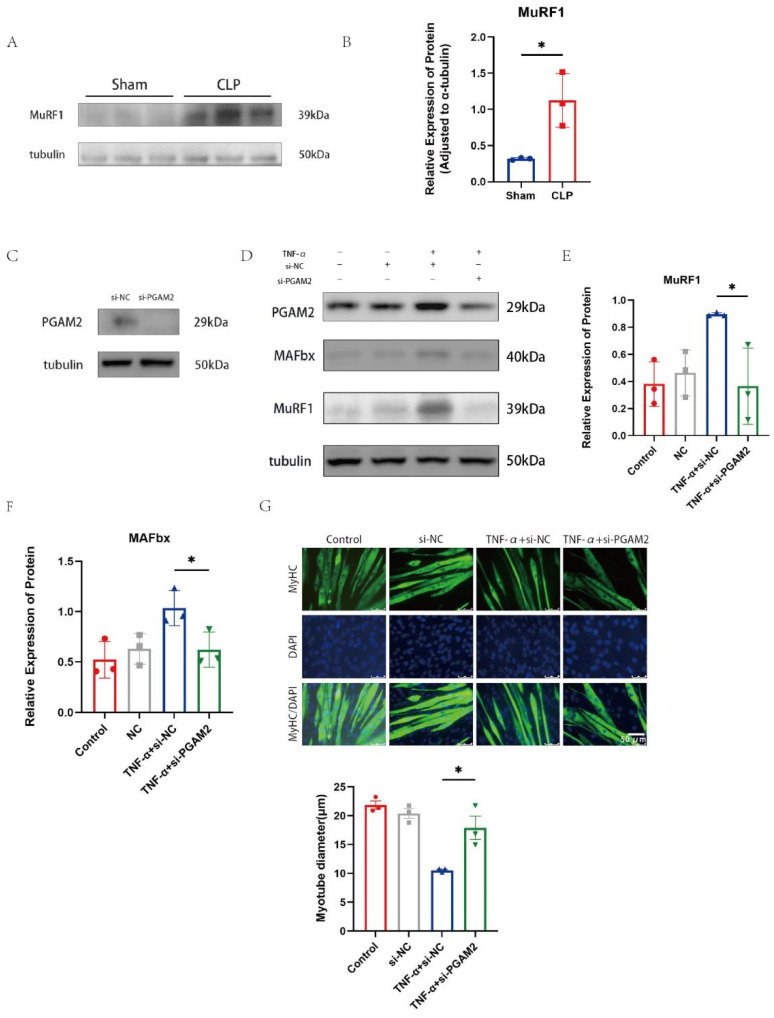
PGAM2 knockdown attenuates TNF-α–induced activation of muscle atrophy–related E3 ligases and myotube atrophy. (**A**,**B**) CLP surgery significantly increased the protein levels of MuRF1 in diaphragm tissue. (**C**) Verification of PGAM2 silencing efficiency in C_2_C_12_ myotubes transfected with siRNA targeting PGAM2. (**D**–**F**) PGAM2 depletion markedly attenuated the CLP- or TNF-α-induced up-regulation of MAFbx and MuRF1 protein expression. (**G**) Representative immunofluorescence images of myotubes stained for myosin heavy chain (MyHC; green) and counterstained with DAPI (blue) to visualize nuclei. Scale bar = 50 µm. * *p* < 0.05.

MAFbx and MuRF1 have been shown in previous studies to be able to reduce stress through the ubiquitin proteasome system (UPS) [26]. Muscle protein degradation is a key process contributing to muscle atrophy, and the ubiquitin–proteasome pathway plays an important role in this process. In the CLP-induced sepsis model, MuRF1 protein levels were significantly increased compared with the sham group (Figure 3A,B). To explore the relationship between PGAM2 and these ligases, PGAM2 expression was silenced in C_2_C_12_ myotubes using small interfering RNA (si-PGAM2). Four groups were established: Control, si-NC (non-targeting siRNA), TNF-α + si-NC, and TNF-α + si-PGAM2. Western blotting verified effective PGAM2 knockdown (Figure 3C,D). TNF-α treatment markedly elevated MAFbx and MuRF1 expression in the si-NC group, whereas PGAM2 silencing attenuated this increase (Figure 3E,F).

Immunofluorescence staining for MyHC was used to evaluate myotube morphology. TNF-α exposure caused obvious myotube thinning and atrophy, while PGAM2 knockdown partially reversed these morphological changes, resulting in larger myotube diameters (Figure 3G). These experimental results indicate that PGAM2 regulates muscle specific E3 ligases (MuRF1 and MAFbx) under inflammatory conditions, and inhibition of PGAM2 can alleviate TNF-α-induced myotube atrophy.

### 3.4. PGAM2 Overexpression Exacerbates TNF-α-Induced Muscle Atrophy

**Figure 4:**
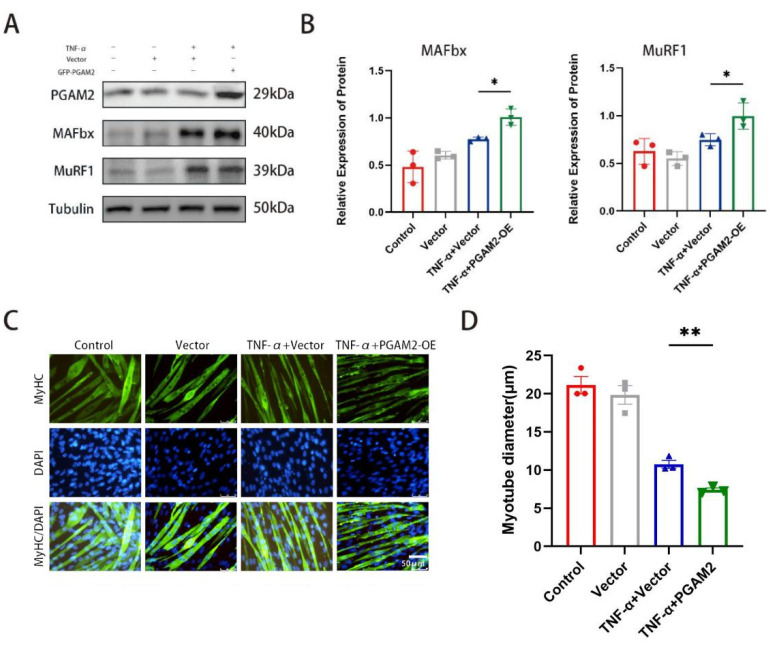
In vitro gain-of-function experiments show that PGAM2 overexpression enhances TNF-α–induced activation of atrophy-related E3 ligases and aggravates myotube atrophy. (**A**,**B**) Representative immunoblots and densitometric quantification showing that PGAM2 overexpression significantly increased the TNF-α-induced elevation of MAFbx and MuRF1 protein levels. (**C**) Representative immunofluorescence micrographs of myotubes labeled with anti-myosin heavy chain (MyHC: green) and counterstained with DAPI (blue) to visualize nuclei. Scale bar = 50 μm. (**D**) Quantitative analysis of myotube diameter across experimental groups. * *p* < 0.05, ** *p* < 0.01.

By constructing and transferring the PGAM2 plasmid, we further investigated PGAM2 overexpression and TNF-α stimulation. The effect of stimulation on myotube formation, myotube diameter, and expression of muscle atrophy related proteins. Our results showed that overexpression of PGAM2 exacerbated muscle atrophy caused by TNF-α (Figure 4A,B). Immunofluorescence staining of MyHC showed that the control and vector groups maintained normal myotube morphology, whereas TNF-α treatment led to visible atrophy. However, there were signs of atrophy in the myotubes of the TNF-α + Vector group, while the degree of atrophy was exacerbated in the TNF-α + PGAM2-OE group (Figure 4C). Quantitative analysis confirmed that myotube diameters were significantly smaller in the TNF-α + PGAM2-OE group than in the other groups (Figure 4D), indicating that PGAM2 overexpression promotes TNF-α-induced muscle atrophy.

### 3.5. PGAM2 Promotes TNF-α-Induced Muscle Atrophy Through Activation of the JAK2/STAT3 Signaling Pathway

Based on the above findings, PGAM2 was identified as a key factor involved in TNF-α-induced muscle atrophy. We hypothesized that PGAM2 exerts its damaging effects on muscle cells by activating an atrophy-related signaling pathway. Previous studies have reported that another PGAM isoform, PGAM1, regulates the JAK2/STAT3 signaling cascade; therefore, we speculated that PGAM2 might also participate in this pathway.

**Figure 5:**
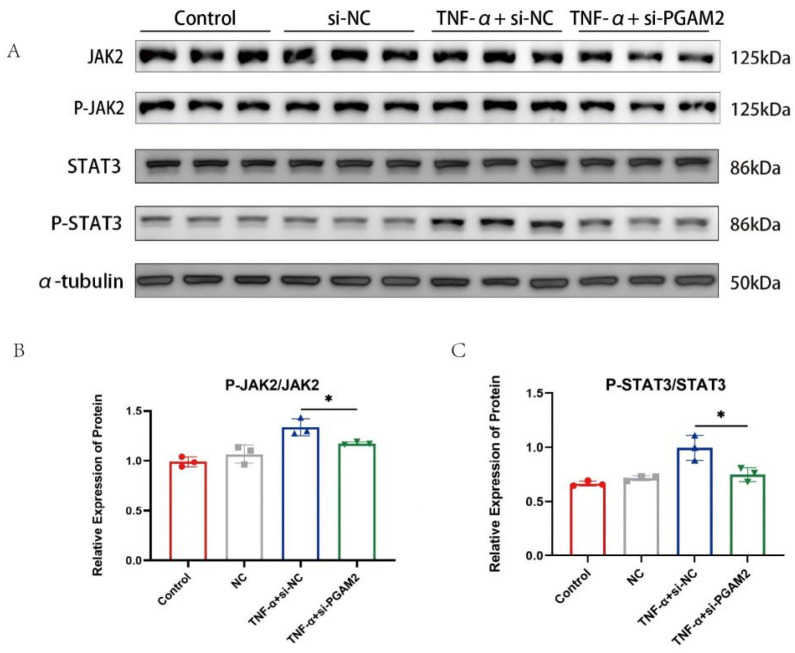
(**A**–**C**) PGAM2 knockdown significantly reduced the CLP-induced phosphorylation of JAK2 and STAT3. * *p* < 0.05.

**Figure 6:**
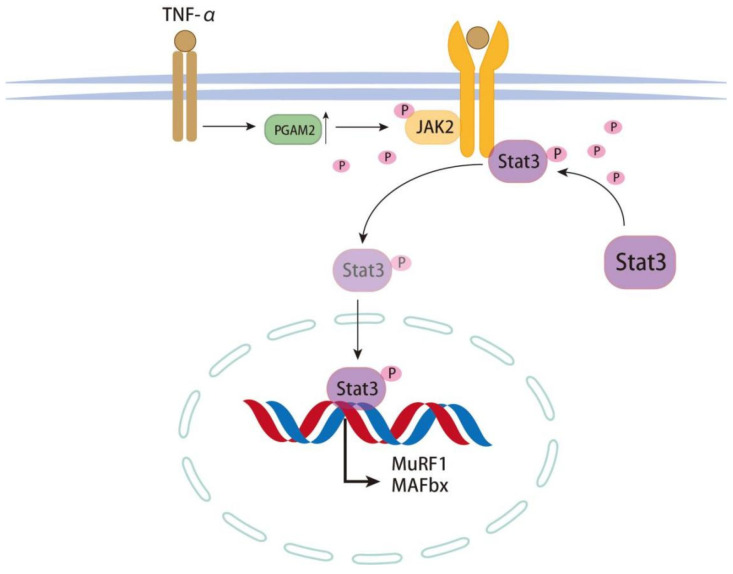
Mechanism of PGAM2 regulating JAK2/STAT3 signaling activation during sepsis.

The JAK2/STAT3 signaling pathway plays a role. Compared with the Sham group, CLP treatment activated the JAK2/STAT3 signaling pathway (Figure 5A,B). Next, to evaluate the role of PGAM2 in regulating the JAK2/STAT3 pathway, C_2_C_12_ myotubes were treated with TNF-α after PGAM2 knockdown. Western blot analysis showed that the levels of phosphorylated JAK2 and STAT3 were markedly reduced in the TNF-α + si-PGAM2 group compared with the TNF-α + si-NC group (Figure 5B,C). These results demonstrate that PGAM2 positively regulates the JAK2/STAT3 signaling pathway and contributes to TNF-α-induced muscle atrophy. A mechanism diagram illustrating the regulatory relationship between PGAM2 and the JAK2-STAT3 signaling pathway is provided in Figure 6.

## 4. Discussion

### 4.1. Main Findings

This study identifies PGAM2 as a key regulator of sepsis-induced diaphragmatic atrophy. We demonstrated that PGAM2 expression was significantly elevated in septic mice and in C_2_C_12_ myotubes treated with TNF-α. Functional studies revealed that PGAM2 knockdown attenuated TNF-α-induced myotube atrophy, whereas PGAM2 overexpression aggravated it. Mechanistically, PGAM2 knockdown reduced the activation of the JAK2/STAT3 signaling pathway, suggesting that PGAM2 may participate in inflammatory atrophy-related signaling. Together, these findings suggest that PGAM2 may act as a mediator linking inflammatory stress, metabolic remodeling, and respiratory muscle atrophy during sepsis.

### 4.2. Relation to Previous Studies

Our results align with recent studies emphasizing the importance of the PGAM family in sepsis. PGAM2 dephosphorylation has been implicated in mitochondrial dysfunction and myocardial injury, and loss of PGAM2 was shown to protect against sepsis-induced cardiac damage [24]. PGAM2, though less studied, was reported to be downregulated in TWEAK-deficient mice, a model resistant to muscle wasting [27]. Our observation of increased PGAM2 expression in septic diaphragms therefore complements existing evidence and suggests that PGAM2 may be differentially regulated across tissues and disease contexts.

Diaphragmatic dysfunction during sepsis remains an important but complex issue, with debate over whether atrophy occurs early or later during disease progression. Clinical studies using ultrasound have demonstrated reduced thickening fraction, contraction velocity, and motion amplitude, suggesting that impaired diaphragmatic performance is a common feature of sepsis [28,29]. Experimental models similarly report decreased contractility linked to calcium handling defects, reduced receptor expression, oxidative stress, and mitochondrial injury [30,31]. Our results extend these observations by providing histological and molecular evidence that PGAM2 is associated with sepsis-induced atrophy of diaphragm fibers.

### 4.3. Mechanistic Insights and Implications

Traditionally, PGAM2 has been characterized as a glycolytic enzyme supporting myoblast differentiation and growth. For instance, it enhances glycolytic flux to promote myofiber fusion and is activated by eicosatetraenoic acid to stimulate skeletal muscle development through the PI3K/AKT pathway [32]. At first glance, these anabolic roles appear inconsistent with our finding that PGAM2 promotes atrophy under septic conditions. However, sepsis-induced muscle wasting is not simply a state of reduced metabolism. Instead, sepsis is accompanied by profound metabolic remodeling, including mitochondrial dysfunction, altered substrate utilization, impaired oxidative phosphorylation, and a compensatory or maladaptive shift toward glycolysis [33,34]. This metabolic shift resembles a Warburg-like pattern, in which increased glycolytic activity does not necessarily indicate anabolic growth but may reflect stress adaptation under inflammatory and hypoxic conditions [35]. Therefore, PGAM2 upregulation in septic diaphragm muscle may represent a maladaptive glycolytic response rather than a growth-promoting signal.

Moreover, enhanced glycolytic enzyme expression may coexist with muscle atrophy because inflammatory signaling, ubiquitin–proteasome activation, mitochondrial energetic failure, and impaired protein homeostasis can override any potential anabolic effect of increased glycolytic activity [36]. In this context, PGAM2 may not only reflect altered energy metabolism but may also participate in atrophy-related signaling under septic stress. This interpretation is also consistent with the broader concept that metabolic enzymes may exert non-canonical functions beyond their classical catalytic roles under pathological conditions [37].

### 4.4. PGAM2 and JAK2/STAT3 Signaling

One important finding of this study is that PGAM2 knockdown attenuated TNF-α-induced myotube atrophy and reduced the phosphorylation of JAK2 and STAT3. The JAK2/STAT3 pathway is widely recognized as an important mediator of inflammatory responses, organ injury, and immune dysregulation in sepsis. Activation of this pathway has also been linked to skeletal muscle wasting through the regulation of inflammatory and proteolytic programs. PGAM has been reported to bind 14-3-3ζ and BCL-xL, exerting anti-apoptotic effects [22], and to interact with STAT3 to regulate IL-6/JAK2/STAT3 signaling [23]. These studies support a context-dependent role for PGAM2, shifting from pro-growth under physiological conditions to maladaptive signaling under inflammatory stress. One important finding of this study is that PGAM2 knockdown attenuated TNF-α-induced myotube atrophy and reduced the phosphorylation of JAK2 and STAT3. The JAK2/STAT3 pathway is widely recognized as an important mediator of inflammatory responses, organ injury, and immune dysregulation in sepsis. Activation of this pathway has also been linked to skeletal muscle wasting through the regulation of inflammatory and proteolytic programs. Our data suggest that PGAM2 may contribute to sepsis-related muscle atrophy, at least partly, through the activation of JAK2/STAT3 signaling.

Our most significant finding is that PGAM2 promotes TNF-α–induced myotube atrophy by activating JAK2/STAT3 signaling. This pathway is widely recognized as a mediator of sepsis pathophysiology, contributing to inflammation, endothelial dysfunction, and immune suppression. Inhibition of JAK2/STAT3 in sepsis models improves survival and reduces organ injury. We show here that PGAM2 knockdown reduced phosphorylation of JAK2 and STAT3, attenuating pathway activation and alleviating atrophy. While the exact mechanism by which PGAM2 regulates JAK2/STAT3 remains unclear, one possibility is direct protein–protein interaction, analogous to PGAM1–STAT3 binding [23]. This provides a mechanistic link between a glycolytic enzyme and inflammatory transcriptional programs in sepsis.

### 4.5. Limitations

This study has several limitations. Although TNF-α has been reported to increase in CLP-induced sepsis models, this response is often transient and varies with the timing and severity of the model [38]. TNF-α was used in our in vitro experiments to mimic inflammatory stimulation, TNF-α mRNA expression in diaphragm tissue was not directly measured in the present study. This limits our ability to directly define the upstream inflammatory regulation of PGAM2 in septic diaphragm muscle. Future studies should assess TNF-α and other inflammatory mediators in diaphragm tissue to further clarify the upstream signals responsible for PGAM2 upregulation.

Second, some molecular analyses were performed with relatively small sample sizes because of limited remaining diaphragm tissue and sample-quality issues after processing. In particular, tissue consumption during previous assays, failed frozen section preparation, poor section quality, or insufficient remaining tissue for molecular assays resulted in slightly different sample numbers among analyses. Given the inherent biological variability of the CLP model, these molecular findings should be interpreted cautiously and require further validation with larger sample sizes.

Third, this study mainly relied on histological and molecular markers of diaphragmatic atrophy. Direct physiological assessments of diaphragm function, such as ex vivo diaphragmatic force production, respiratory mechanics, or in vivo contractile performance, were not performed. Although diaphragm weight, muscle fiber cross-sectional area, and atrophy-related molecular markers provide evidence of diaphragmatic injury, future studies incorporating direct functional measurements are needed to better establish the physiological and clinical relevance of PGAM2-mediated diaphragmatic dysfunction in sepsis.

Fourth, although we discussed the potential relationship between PGAM2 upregulation and sepsis-induced metabolic remodeling, we did not directly assess glycolytic flux, lactate production, mitochondrial respiration, or mitochondrial ultrastructure. Therefore, whether PGAM2 upregulation represents a compensatory metabolic adaptation or a maladaptive glycolytic response remains to be further determined. Future studies combining metabolic flux analysis, mitochondrial functional assays, and genetic models are needed to clarify the metabolic role of PGAM2 in septic diaphragm muscle.

### 4.6. Future Directions

Further research is needed to explore the dual role of PGAM2 in muscle physiology and pathology. While PGAM2 supports differentiation and growth under normal conditions, it appears to drive atrophy in sepsis. This functional divergence may reflect differential metabolic reprogramming, whereby PGAM2 modulates glycolysis and mitochondrial activity in response to inflammatory cues. Multi-omics approaches, including transcriptomics, proteomics, and metabolomics, could map the broader regulatory network of PGAM2 in sepsis. Additionally, crosstalk between PGAM2 and other signaling cascades, such as PI3K/AKT, warrants investigation. Elucidating these pathways may identify PGAM2 as both a biomarker and a therapeutic target for sepsis-related respiratory muscle dysfunction.

## 5. Conclusions

In summary, this study demonstrates that PGAM2 is upregulated in septic diaphragms and TNF-α–treated myotubes. PGAM2 promotes muscle atrophy by activating the JAK2/STAT3 signaling pathway and regulating atrophy-related proteins (MAFbx and MuRF1). These findings establish PGAM2 as a novel mediator of sepsis-induced diaphragmatic atrophy and suggest that targeting PGAM2 may provide therapeutic benefit.

## Figures and Tables

**Table 1:** Primers used for RT-qPCR.

Gene	Forward (5′-3′)	Reverse (3′-5′)
β-actin	CATCCGTAAAGACCTCTATC	ATGGAGCCACCGATCCACA
MAFbx1	GAGGCAGATTCGCAAGCGTT	TCCAGGAGAGAATGGCAGT
MuRF1	AGTGTCCATGTCTTGGAGGT	ACTGGAGCACTCCTGCTTGT
PGAM2	ATGGAAGGAGGAAGTGAGG	GGTGGTCTCCTCGGAGTCAA
IL-1β	TGGGATGATTCCATGAGCTT	CTTGGTGGTGGTGGTGGAAG
IL-6	GGGACTGATGCTGGTGACAA	CGCACTAGGTTTGCCGAGTA

## Data Availability

The original contributions presented in this study are included in the article. Further inquiries can be directed to the corresponding author.

## Abbreviations

The following abbreviations are used in this manuscript:

PGAM2	Multidisciplinary Digital Publishing Institute
CLP	Cecal Ligation and Puncture
C57BL/6 mice	C57BL/6 mouse strain
TNF-α:	Tumor necrosis factor alpha
JAK2	Janus kinase 2
STAT3	Signal transducer and activator of transcription 3
UPS	Ubiquitin–proteasome system
CSA	Cross-sectional area
MyHC	Myosin heavy chain
IL-1β	Interleukin-1β
IL-6	Interleukin-6
mTOR	Mammalian target of rapamycin
IKKα	Inhibitor of nuclear factor kappa-B kinase subunit alpha
HSP90	Heat shock protein 90
SYVN1	Synovial apoptosis inhibitor 1
BAD	BCL2-associated agonist of cell death
BCL-xL	B-cell lymphoma-extra large
PI3K/AKT	Phosphatidylinositol 3-kinase/protein kinase B
